# Differential effects of plant-based flours on metabolic homeostasis and the gut microbiota in high-fat fed rats

**DOI:** 10.1186/s12986-023-00767-8

**Published:** 2023-10-19

**Authors:** Taylor M. Martinez, Hallie R. Wachsmuth, Rachel K. Meyer, Savanna N. Weninger, Adelina I. Lane, Archana Kangath, Gabriele Schiro, Daniel Laubitz, Jennifer H. Stern, Frank A. Duca

**Affiliations:** 1 Physiological Sciences Graduate Interdisciplinary Program, University of Arizona, Tucson, AZ USA; 2 School of Nutritional Science and Wellness, University of Arizona, Tucson, AZ USA; 3 School of Animal and Comparative Biomedical Sciences, University of Arizona, ACBS Building, 1117 E Lowell St., Tucson, AZ 85711 USA; 4 The PANDA Core for Genomics and Microbiome Research, Department of Pediatrics, University of Arizona, Tucson, AZ USA; 5 Division of Endocrinology, University of Arizona College of Medicine, Tucson, AZ USA; 6 BIO 5 Institute, University of Arizona, Tucson, AZ USA

**Keywords:** Obesity, Microbiome, Metabolism, Fiber

## Abstract

**Background:**

The gut microbiome is a salient contributor to the development of obesity, and diet is the greatest modifier of the gut microbiome, which highlights the need to better understand how specific diets alter the gut microbiota to impact metabolic disease. Increased dietary fiber intake shifts the gut microbiome and improves energy and glucose homeostasis. Dietary fibers are found in various plant-based flours which vary in fiber composition. However, the comparative efficacy of specific plant-based flours to improve energy homeostasis and the mechanism by which this occurs is not well characterized.

**Methods:**

In experiment 1, obese rats were fed a high fat diet (HFD) supplemented with four different plant-based flours for 12 weeks. Barley flour (BF), oat bran (OB), wheat bran (WB), and Hi-maize amylose (HMA) were incorporated into the HFD at 5% or 10% total fiber content and were compared to a HFD control. For experiment 2, lean, chow-fed rats were switched to HFD supplemented with 10% WB or BF to determine the preventative efficacy of flour supplementation.

**Results:**

In experiment 1, 10% BF and 10% WB reduced body weight and adiposity gain and increased cecal butyrate. Gut microbiota analysis of WB and BF treated rats revealed increases in relative abundance of SCFA-producing bacteria. 10% WB and BF were also efficacious in preventing HFD-induced obesity; 10% WB and BF decreased body weight and adiposity, improved glucose tolerance, and reduced inflammatory markers and lipogenic enzyme expression in liver and adipose tissue. These effects were accompanied by alterations in the gut microbiota including increased relative abundance of *Lactobacillus* and *LachnospiraceaeUCG001*, along with increased portal taurodeoxycholic acid (TDCA) in 10% WB and BF rats compared to HFD rats.

**Conclusions:**

Therapeutic and preventative supplementation with 10%, but not 5%, WB or BF improves metabolic homeostasis, which is possibly due to gut microbiome-induced alterations. Specifically, these effects are proposed to be due to increased concentrations of intestinal butyrate and circulating TDCA.

**Supplementary Information:**

The online version contains supplementary material available at 10.1186/s12986-023-00767-8.

## Introduction

The prevalence of obesity continues to rise, with estimates that 42% of the U.S. adult population will be obese by the year 2030 [1, 2]. This alarming increase in obesity is associated with an increased consumption of a Western Diet, characteristically high in fat and sugar and low in fiber [3, 4]. As consumption of whole-food plant sources has declined, less than 8% of adults in the U.S. consume the daily recommended dietary fiber values [5]. Conversely, fiber supplementation improves body weight, adiposity, waist circumference, and serum glucose and insulin levels [6]. Thus, a possible solution to address the rising levels of obesity in the U.S is via dietary modifications to increase fiber consumption.

Obesity and diabetes are both associated with unique gut microbiome profiles, and alterations in the gut microbiome have been implicated in the success of many metabolic therapeutics [7–9]. The diet is the predominant regulator of gut microbiota composition [10–12]. In the case of dietary fiber, increased fiber consumption is associated with shifts in the gut microbiota [6, 13, 14]. Specifically, increased fiber consumption results in increased abundance of short-chain fatty acid (SCFA)-producing taxa such as *Roseburia*, *Bifidobacteria*, and *Akkermansia* [15–20], which are inversely correlated with obesity and associated with improvements in gut permeability and reductions in metabolic endotoxemia, both characteristics of obesity [18, 19, 21, 22]. However, dietary fiber is extremely heterogenous, and little is known about how various dietary fibers differentially impact the gut microbiome and subsequently host health.

Plant-based flours vary in fiber solubility, viscosity, and palatability, all of which impact their function and effects on the gut microbiome. For example, Hi-maize amylose (HMA) is high in resistant starch with mixed solubility, whereas insoluble wheat bran (WB) contains wheat dextrin fiber and both barley flour (BF) and oat bran (OB) are composed of soluble viscous β-glucan. While many studies have independently investigated plant-based flours on metabolic parameters, many of which have found beneficial effects on metabolic homeostasis [23–28], a comprehensive and comparative analysis of these flours has yet to be conducted. In the following study, four plant-based flours (HMA, WB, BF, OB) differing in solubility and fiber composition were tested for their therapeutic potential to improve diet-induced obesity. Given that many of the previous work examining these plant-based flours demonstrated changes in the gut microbiota, we examined shifts in the gut microbiota as well as changes in SCFAs and bile acids, both gut-derived metabolites known to impact metabolic homeostasis. We hypothesized that while the same plant-based flours at low (5%) or high (10%) supplementation would result in similar shifts in specific bacterial taxa in the gut microbiota, the high supplementation (10%) would more robustly shift the gut microbiota and increase SCFA production due to a greater amount of substrate for the bacteria to utilize, leading to a greater impact on metabolic homeostasis. Further, we hypothesized that rather than solubility or viscosity of the dietary flours (HMA, WB, BF, and OB), the specific fibers composition within the flour would contribute more robustly to gut microbiome shifts and metabolic improvements, with flours high in β-glucan (BF and OB) and resistant starch (HMA) being most effective based on previous studies [6].

## Materials and methods

### Rats

8-week-old male Sprague–Dawley rats were purchased from Charles River Laboratories (Wilmington, MA). Rats were single housed and maintained on a 12-h light/dark cycle with ad libitum access to a chow (Research Diets D12450H) diet and water prior to the start of the treatment diets. All rats were housed and maintained in accordance with the University of Arizona Institutional Animal Care and Use Committee (IACUC).

### High fat and fiber diet treatment

In the following study, rats were maintained on either a macronutrient-matched chow (Research Diets), HFD (Research Diets D42151) or specially formulated HFD supplemented with either HMA, WB, BF, or OB at either 5% or 10% fiber content by weight (Research Diets D20011001-7, Additional file 2: Table S1). The fiber diets were macronutrient matched to the HFD (with similar but not identical kilocalories in some instances) with the fiber component of the flour replacing cellulose, and the other carbohydrates in the flour replacing the corn starch in the HFD (D42151). However, a 10% OB diet could not be created due to an inability to macronutrient-match the diet.

In Experiment 1, 2 cohorts (n = 5–6 rats per group per cohort) of 10wk old rats with an average weight of 363 ± 30 g, were single-house, placed on a HFD, and allowed to eat ad libitum for 5 weeks to induce obesity, defined as a significant increase in adiposity compared to healthy chow-fed rats. After the 5 weeks, the rats weighed on average 562 ± 55 g and had an average adiposity of 14 ± 4.8%, significantly increased compared to chow fed controls (Additional file 1: Figure S1A-C). Rats were then switched to HFD + flour diets and allowed to eat ad libitum for 12 weeks (n = 10–12/diet group from 2 separate cohorts of 5–6 rats per group).

For Experiment 2, 10wk old rats with an average weight of 450 ± 23 g and average adiposity of 6 ± 2% were single-housed and placed on either the HFD, 10% WB, or 10% BF supplemented diet for 12 weeks without inducing obesity prior (n = 12/group). Three weeks after starting dietary treatment, rats were individually housed in metabolic cages (Promethion Core, Sable Systems) for 3 days with 48 h for acclimation, and the final 24 h for recordings. Indirect calorimetry was utilized to measure energy expenditure and RER, and food intake. Data were converted using the ExpeData and Macro Interpreter program and analyzed with GraphPad Prism software.

In both experiments, body weight was recorded every week and body fat percentage was determined every 3 weeks via imaging with EchoMRI™-500 Body Composition Analyzer (EchoMRI, Houston, TX). For experiment 1, body weight and adiposity data are expressed as percent change from week 0 to accurately represent the effects of dietary treatment from two separate cohorts; percent change was calculated with the following formula: ((week X—week 0)/week 0)*100. At the end of both experiments, rats were 4 h fasted and deeply anesthetized and blood (portal plasma) and tissue samples (liver and epididymal adipose) were harvested for analysis, immediately followed by animal euthanasia.

### Cecal microbiota analysis

Individual cecal microbiota were assessed based on the V4 fragment of 16S rRNA gene, as published previously [29, 30]. As previously described, samples were mechanically disrupted with the TissueLyser II (MO BIO Laboratories) and total DNA from the cecal samples were purified using the QIAamp PowerFecal Pro DNA Kit (Qiagen) according to manufacturer protocol. No template controls were used to control for potential contamination during the extraction and sequencing procedure. All samples were pooled into a sequencing library, as previously published [29, 30]. A 7.25 pM library was sequenced at Microbiome Core at the University of Arizona Steele Children’s Research Center on MiSeq platform (Illumina) using custom primers [31]. Demultiplexing was performed using idemp (https:// github.com/yhwu/idemp). Quality filtering, error correction, dereplication, chimera identification, and merging of paired-end reads were performed using the DADA2 pipeline [32]. The ASVs taxonomy was assigned using the Ribosomal Database Project (RDP) classifier [33] against SILVA database (release 138). Richness was calculated on rarefied data (10,920 reads), with significant differences tested with a nonparametric Kruskal–Wallis test followed by a Dunn’s post hoc test (*p* < 0.05 after FDR correction). Compositional differences between sample groups were tested using a PERMANOVA analysis based on Bray–Curtis dissimilarities, using the function “adonis” of the vegan R package. Non-metric multidimensional scaling (NMDS) ordination were used to visualize such differences. ﻿A nonparametric Kruskal–Wallis test followed by a Dunn’s post hoc test (*p* < 0.05 after FDR correction) was performed to examine if the relative abundances of genera of interest were significantly different among sample groups. DeSeq2 package [34] was further used to calculate differential abundance between experimental groups at phylum level.

### Short chain fatty acid analysis

Cecal contents were sent to Metabolon Inc. for SCFA extraction via bead beating and subsequent measurement of SCFA concentrations on a high-performance liquid chromatography (LC)-tandem mass-spectrometry (MS/MS) platform as previously described [35]. SCFA concentrations were measured and presented per gram of cecal contents analyzed.

### Cytokine and lipogenic enzyme PCR

Real-Time qPCR for adipose and liver was performed as previously described [30]. TaqMan™ Gene Expression Assays (ThermoFischer Scientific) for *Tnf* (tumor necrosis factor-α) (Rn99999017_m1) and *Il6* (interlukin-6) (Rn01410330_m1) were used to measure cytokine expression and *Fasn* (fatty acid synathase) (Rn00569117_m1), *Pparg* (peroxisome proliferator-activated receptor -γ) (Rn00440945_m1), *Scd1* (stearoyl-Coenzyme A desaturase) (Rn06152614_s1), *Acc* (acetyl Coenzyme A carboxylase) (Rn00573474_m1), *Lpl* (lipoprotein lipase) (Rn00561482_m1) were used to measure lipogenic enzyme expression with *Rps18* (18 s ribosomal RNA) (Rn01428913_gH) used for standardization.

### Western blotting

Liver tissue was collected, crushed, and placed in NP40 lysis buffer with protease and phosphatase inhibitor for homogenization. Homogenized samples were centrifuged at 12,000 rpm for 15 min at 4 °C, and the supernatant was collected. A BCA protein assay kit (ThermoScientific Cat#: 23,225) was used to determine total protein content of the samples, per manufacturer protocols. 20ug of denatured protein was loaded into a 10% Criterion TGX precast gel (Bio-Rad Cat#: 5,671,033) where they were separated by electrophoresis and transferred to a PVDF membrane using the wet transfer system. Membrane was blocked in 5% bovine serum albumin and incubated overnight at 4 °C with the primary antibodies fatty acid synthase (1:500, Cat# ab22759, Abcam), acetyl CoA carboxylase (1:500, Cat# 3662, Cell Signaling), lipoprotein lipase (1:500 Cat# sc-373759, Santa Cruz), stearoyl-Coenzyme A desaturase (1:1000, Cat# ab236868), β-actin (1:5000, Cat# 4970, Cell Signaling), Glyceraldehyde 3-phosphate dehydrogenase (1:2000, Cat# 2118, Cell Signaling). Membrane was washed with TBST, then incubated with horseradish peroxidase conjugated IgG secondary antibodies 1:20,000 (rabbit anti-mouse Cat# ab6728, Abcam; goat anti-rabbit Cat# ab6721, Abcam) for 1 h at room temperature. Protein signals were detected by West Pico Enzyme substrate complex (1:1 ratio) and imaged using the Azure 600 Imager.

### Liver triglyceride analysis

Pointe MedTest DX Triglyceride Liquid Reagent Set (Cat #: T7532-120) was used to isolate and quantify liver triglycerides in a colorimetric assay read at 570 nm as previously described [36, 37] and denoted as mg of triacylglycerol/ g of liver tissue.

### Glucose and insulin tolerance tests

At 12 weeks supplementation, rats underwent a glucose tolerance test, followed 3–5 days later by an insulin tolerance test. Rats were fasted for 6 h prior to the intraperitoneal glucose tolerance test (IPGTT) and insulin tolerance test (ITT). For the IPGTT, rats were injected intraperitoneally with 45% Glucose solution at 1.5 g/kg. Blood glucose measurements were taken via tail vein at 0, 15, 30, 60, 90, and 120 min via handheld glucometer. For the ITT, rats were injected with insulin (0.75 U/kg; Sigma I0516-5ML). Blood glucose measurements were taken via tail vein at 0, 30, 60, 90, and 120 min time points.

### Gut permeability assay

Five days following tolerance tests, rats were subject to a terminal gut permeability test. Following assay and previously published protocols [38], rats (n = 8–10/ group) were fasted for 4 h prior to gavage with 100 mg/ml FITC-dextran (Sigma Aldrich) at 60 mg/100 g of body weight. Rats were sacrificed 4 h later as described above, and blood was collected via portal vein. Plasma fluorescence was read compared to FITC-dextran standards on Molecular Devices Spectramax M5 plate reader.

### Endotoxin measurements

Portal plasma was collected by endotoxin-free pipette tips (Finntip™ Filtered Pipette Tips, ThermoFisher Scientific) and stored in endotoxin-free tubes (ThermoFisher Scientific) at -80 °C prior to analysis. Plasma samples were thawed, diluted 1:50 in Endotoxin-free water (from kit), and heat shocked at 70 °C for 15 min prior to analysis with Pierce™ Chromogenic Endotoxin Quant Kit (ThermoFisher Scientific). Samples were plated in individually wrapped endotoxin free 96-well flat bottom plates (Endosafe, Charles River; product code M9005) according to kit protocol and the plate was analyzed.

### GLP-1 and PYY elisa

Millipore GLP-1 Total ELISA kit (Cat # EZGLP1T-36 K—sensitivity: 1.5 pM) and Aviva Systems Biology PYY ELISA kit (Cat # OKEH04432—sensitivity: 8.14 pg/mL) was used to analyze the portal plasma concentrations after fasting at the end of experiment 1. Samples where the absorbance did not fall within the ELISA standard curve range or where the coefficient of variability was above 10% were removed. ELISAs were run according to manufacturer guidelines and were read at 450 nm and 590 nm (GLP-1) and 450 nm (PYY) to determine pM gut peptide concentrations.

### Bile acid analysis

Portal plasma bile acids were analyzed at the University of Arizona Cancer Center Analytical Chemistry Shared Resource via liquid–liquid extraction utilizing ethyl acetate as previously described [39].

### Statistical analysis

Statistical analyses were completed using GraphPad Prism 8 software (GraphPad Software). Normality was analyzed in GraphPad Prism 8 prior to statistical analyses. The D’Agostino-Pearson omnibus K2 normality test was used when applicable, the Shapiro–Wilk normality test was used on data with small sample sizes (adiposity data). All data other than SCFA data passed normality tests. Body weight and change in adiposity were analyzed using a two-way ANOVA with repeated measures and a Dunnett’s post hoc analysis. To assess the effect of diet (HFD, BF, and WB) on energy expenditure and food intake (light cycle, dark cycle, and total 24 h), we used the mixed model procedure in SAS Enterprise Guide 7.1 (SAS Institute Inc., Cary, NC), including lean mass as a covariate (ANCOVA). Difference between means was assessed by post-hoc analysis using the Tukey–Kramer correction for multiple comparisons. Glucose/insulin tolerance tests were analyzed using two-way ANOVA with multiple comparisons with Tukey’s post hoc analysis. Metabolic cage data, gut peptides, AUC, bile acids, liver triglycerides, inflammatory cytokines, and endotoxin measurements were analyzed using a one-way ANOVA with multiple comparisons with Tukey’s post hoc analysis. Simple linear regression analyses were run on lipogenic enzymes and liver triglycerides. SCFA data were analyzed using a Kruskal–Wallis Test for non-parametric data Spearman correlation for SCFAs and taxa at the genus level was calculated. *p* < 0.05 was considered significant. Data presented as mean ± SEM.

## Results

### Experiment 1

#### Wheat bran and barley flour supplementation attenuate adiposity gain

**Fig. 1:**
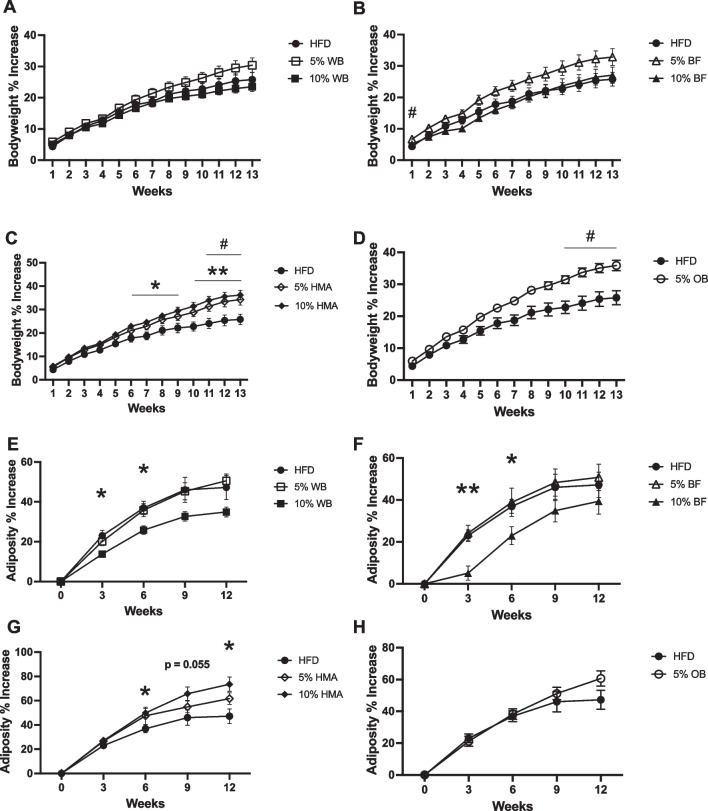
Effect of flour supplementation on body weight and adiposity in obese rats. Body weight percent change in rats fed a HFD or a HFD supplemented with 5% or 10% WB (**A**), BF (**B**), HMA (**C**), or 5% OB (**D**). Percent change in adiposity in the same rats, 5% or 10% WB (**E**), BF (**F**), HMA(**G**), or 5% OB (**H**). Data presented as mean ± SEM (n = 10–12 per group); *represents significance between 10% flour and HFD, *p < 0.05, **p < 0.01, ***p < 0.001, # represents significance between 5% flour and HFD, # *p* < 0.05

Five weeks of HFD feeding induced obesity, as determined by a significant increase in body weight and adiposity of the HFD-fed rats compared to semi-purified macronutrient matched chow-fed control rats (Additional file 1: Figure S1A-C). After 5 weeks, rats were switched to the various diets outlined in Additional file 2: Table S1. After 12 weeks of dietary fiber intervention, rats in all diet groups exhibited no reductions in body weight gain compared to HFD rats (Fig. 1A-D), with OB and HMA groups exhibiting increases in body weight gain compared with HFD control rats (Fig. 1C, D). None of the diet groups demonstrated changes in raw body weight or adiposity (Additional file 1: Figure S1D-K). However, both the 10% BF and WB diets decreased adiposity gain of rats compared with HFD at 3 and 6 weeks on the diet, (Fig. 1E, F). The 10% HMA diet increased adiposity gain (Fig. 1G) while the 5% OB diet had no effect on adiposity gain (Fig. 1H). None of the 5% flour diets led to improvements in body weight (Fig. 1A-D) or adiposity (Fig. 1E–H).

### Dietary flour supplementation alters the cecal gut microbiota

**Fig. 2:**
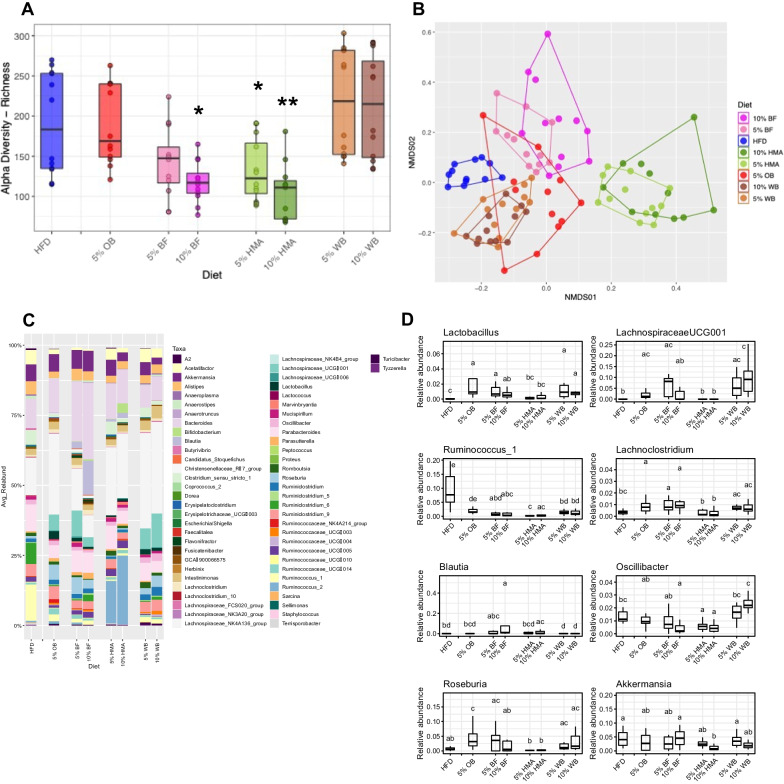
Cecal gut microbiota analysis of flour supplemented rats. Alpha diversity index ASV/Species Richness. Statistically significant differences between all diet groups were determined using a Kruskal–Wallis test followed by a FDR-corrected pairwise Wilcox test; *represents significance between diet groups and HFD (*p < 0.05, **p < 0.01) (**A**). Non-metric multidimensional scaling (NMDS) using Bray–Curtis dissimilarity where each dot represents one animal; ADONIS, permutations = 999, p = 0.001, R^2^ = 0.423 (**B**). Beta diversity of each diet group at the genus level (**C**). Stacked bar plot showing the composition of the microbial community at genus level for each of the analyzed groups (**D**). Boxplots showing relative abundance of selected genera per group. ﻿Letters represent grouping according to Dunn’s Kruskal–Wallis Multiple Comparisons test (significant *p* < 0.05 after FDR correction)

The 5% and 10% HMA and 10% BF groups had decreases in cecal gut microbiota alpha diversity compared with HFD-fed controls (Fig. 2A). Supplementation with each flour significantly shifted the beta diversity and composition of the cecal gut microbiota from HFD control rats (ADONIS, p = 0.001, R2 = 0.423), indicating that the cecal microbiota was influenced by dietary intervention. There were no significant differences in beta diversity and composition of the cecal microbiota between the 5% and 10% flour supplementation groups within the same diet group (Fig. 2B, C). All flour supplemented groups, besides HMA supplemented groups, exhibited increased *Lactobacillus* relative abundance compared with HFD controls. All flour groups except both HMA and 10% BF had increased *LachnospiraceaeUCG001* relative abundance compared with the HFD group. Additionally, all flour supplemented groups, other than OB, had decreased *Ruminococcus_1* relative abundance compared to HFD control. OB and BF supplemented groups had significantly increased *Lachnoclostridium* relative abundance compared to HFD control. 10% BF and 10% HMA groups had increased *Blautia* relative abundance*,* and 10% BF and both HMA groups had decreased *Oscillibacter* relative abundance compared to HFD control. *Roseburia* relative abundance was only increased in rats supplemented with OB compared to all other groups. 10% HMA-fed rats exhibited a reduction in *Akkermansia* relative abundance compared with HFD (Fig. 2D; results of all present genera in Additional file 2: Table S2).

### Dietary plant-based flour supplementation increases SCFA production

**Fig. 3:**
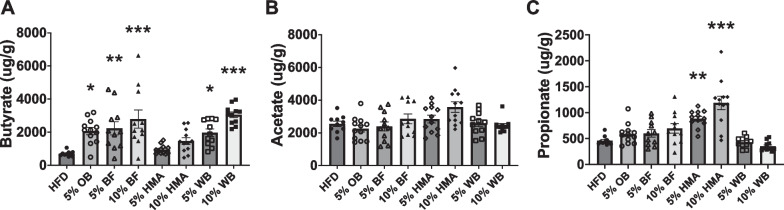
Cecal SCFA levels following dietary flour supplementation. Cecal levels of butyrate (**A**), acetate (**B**), and propionate (**C**) in rats fed a HFD or flour supplemented HFD for 12 weeks. Data presented as ug of SCFA/g of cecal content weight. Mean ± SEM (n = 10–12 per group); *represents significance from HFD control group, *p < 0.05, **p < 0.01, ***p < 0.001

After 12 weeks of flour supplementation, cecal butyrate levels were increased in BF, WB, and OB compared to HFD controls, with a non-significant dose response in the WB (p = 0.13) (Fig. 3A). Cecal acetate levels were unaltered by diet (Fig. 3B), and cecal propionate levels were increased in both HMA supplemented groups (Fig. 3C) compared to the HFD group. Further, we found that some gut microbiota genera were correlated with SCFA levels. Specifically, we found that *Ruminococcus_1* was negatively correlated with cecal levels of butyrate (R^2^ = -0.222, *p* < 0.05) and propionate (R^2^ = -0.404, *p* < 0.0001) and that *Blautia* was positively correlated with cecal butyrate levels (R^2^ = 0.353, *p* < 0.001) (Additional file 2: Tables S3, S4).

### Experiment 2

#### 10% wheat bran and barley flour supplementation prevent obesity through a reduction in energy intake

**Fig. 4:**
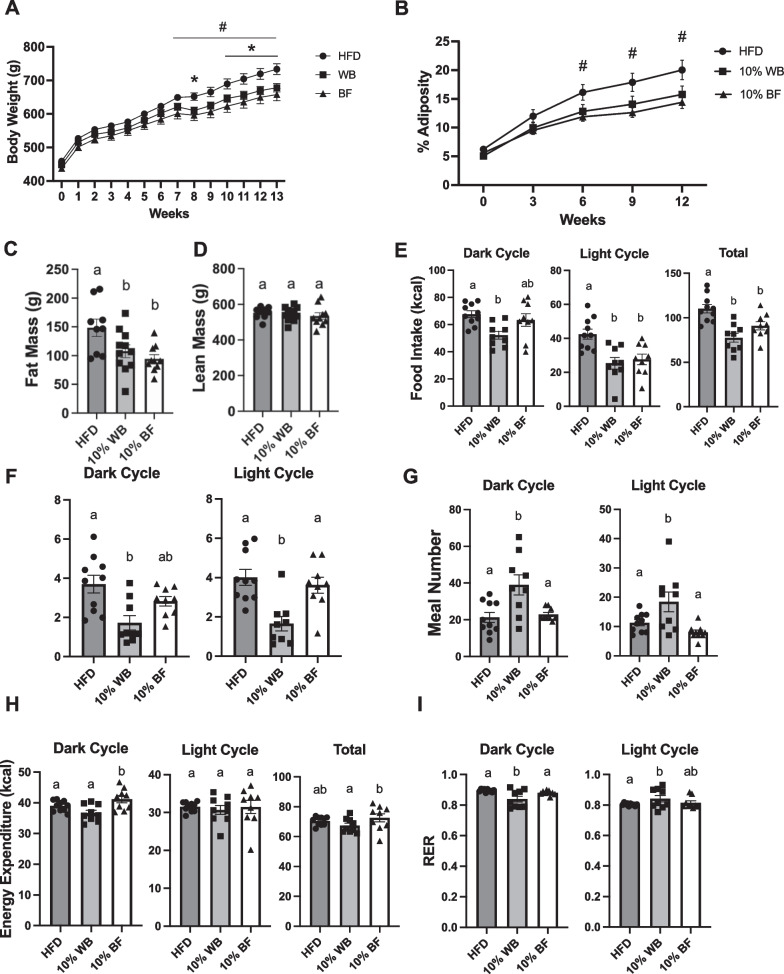
Changes in body weight and adiposity of rats on either a HFD or HFD supplemented with 10% barley flour or wheat bran. Body weight (**A**) and adiposity (**B**) of WB, BF, and HFD-fed rats. Week 12 total fat mass (**C**) and lean mass (**D**). 24-h metabolic cage data at week 3 of dietary intervention; energy intake (**E**), meal size (**F**), meal number (**G**), energy expenditure (**H**), and respiratory exchange ratio (RER) (**I**). Data presented as mean ± SEM (n = 9–10 per group); *p < 0.05 wheat group from HFD, #*p* < 0.05 barley group from HFD; different letters indicate a significant difference between groups *p* < 0.05

In experiment 1, 10% WB and BF supplementation conferred the most metabolic improvements. Therefore, the next experiment tested the effectiveness of these fibers to prevent the development of diet-induced obesity. HFD supplemented with 10% WB or BF led to decreased body weight by the end of the study and decreased adiposity starting at 6 weeks of dietary intervention (Fig. 4A, B) compared to HFD-fed rats. Additionally, at the end of the study, both WB and BF supplementation decreased total fat mass without affecting lean mass (Fig. 4C, D). To determine if WB and BF altered energy expenditure or energy intake to improve energy homeostasis, all groups were placed in metabolic cages at week 3 of dietary intervention, before significant effects on body weight or adiposity were observed. Rats supplemented with both WB and BF had reductions in total caloric intake compared with HFD-fed controls, with both flour supplemented groups demonstrating reductions in caloric intake during the light cycle, while only the 10% WB-fed rats had reductions in the dark cycle (Fig. 4E). The reduction in caloric intake in the rats supplemented with WB was coupled with nearly a 50% reduction in meal size in both the light and dark cycle (Fig. 4F). This was slightly offset by an almost doubling of meal number in dark cycle (Fig. 4G). Interestingly, although the BF supplemented rats demonstrated a similar reduction in total caloric intake as WB supplemented rats, there were no differences in meal size or number in the BF supplemented group (Fig. 4F, G). Neither flour supplemented group demonstrated differences in total energy expenditure compared with the HFD-fed controls, although the BF supplemented group did exhibit increased energy expenditure in the dark cycle (Fig. 4H). WB supplemented rats had a slight decrease in respiratory energy ratio (RER) in the dark cycle and a slight increase in RER in the light cycle compared with HFD-fed control rats (Fig. 4I).

#### Improvements in glucose tolerance and insulin sensitivity

**Fig. 5:**
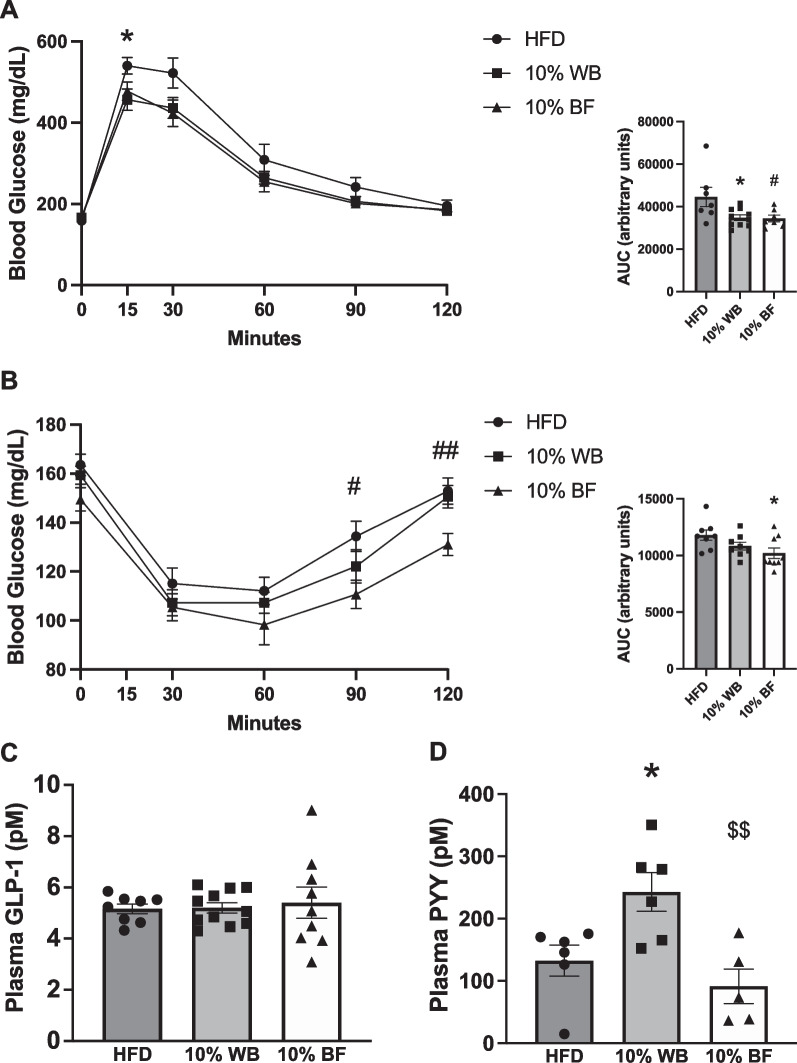
Assessment of glucose homeostasis and gut peptides in flour supplemented rats. Blood glucose concentration and area under the curve (AUC) of an intraperitoneal glucose tolerance test (IPGTT) in HFD-fed and WB and BF supplemented HFD-fed rats (n = 6–10 per group) (**A**). Blood glucose concentration and area under the curve of an ITT (n = 8–9 per group) (**B**). Portal vein active GLP-1 (n = 8–11 per group) (**C**) and PYY (n = 5–6 per group) (**D**) after a 5 h fast. Data presented as mean ± SEM; *indicate significance between WB and HFD, # indicate significance between BF and HFD, and $ indicate significance between WB and BF. *p < 0.05, **p < 0.01, ***p < 0.001 wheat group from HFD, #*p* < 0.05, ##*p* < 0.01 barley group from HFD, and $*p* < 0.05 wheat group from barley group

After 12 weeks of flour supplementation, the WB- and BF-fed groups exhibited improved glucose tolerance compared to HFD-fed rats (Fig. 5A). However, only the 10% BF group had decreased blood glucose at the 90, and 120 min time points and reduced area under the curve compared to HFD following an ITT (Fig. 5B).

#### Wheat bran supplementation increases portal PYY concentrations

Neither WB nor BF groups had differences in portal levels of active GLP-1 compared with HFD (Fig. 5C), however portal PYY concentration was increased in the WB-fed group (Fig. 5D).

#### 10% wheat bran and barley flour effect expression of lipogenic enzymes and proteins and liver triglyceride content

**Fig. 6:**
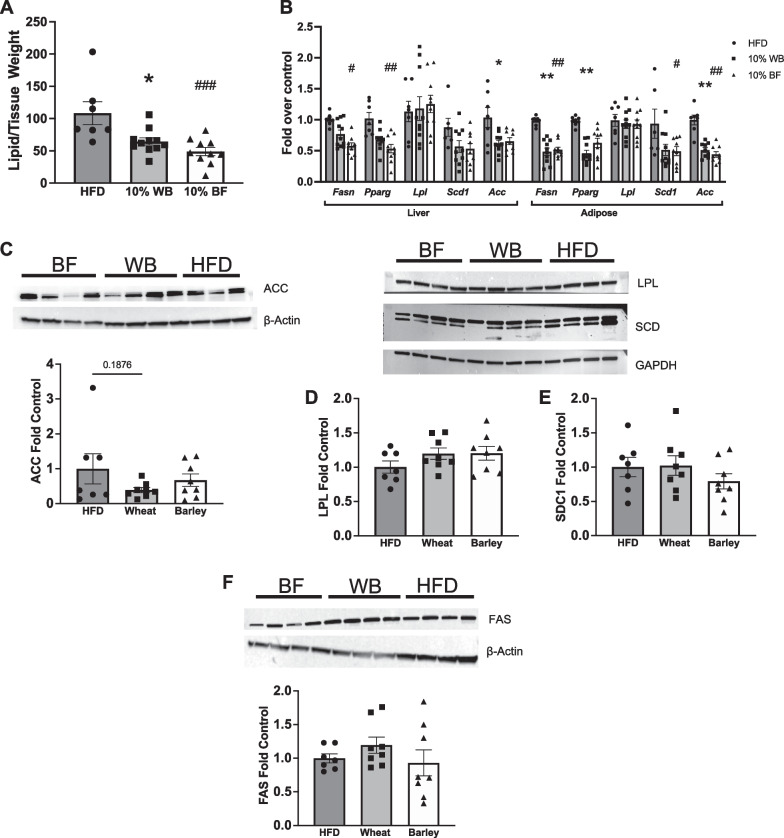
Liver triglycerides and mRNA expression of enzymes involved in lipogenesis in liver and white adipose tissue and western blots of liver enzymes. Liver triglyceride amount by tissue weight (n = 7–11 per group) (**A**). Relative mRNA expression of enzymes involved in lipogenesis in the liver and adipose tissue (n = 6–11 per group) (**B**). Western blots of hepatic ACC (**C**), LPL (**D**), SCD (**E**), and FAS (**F**) quantified as fold control protein expression. Data presented as mean ± SEM; *p < 0.05, **p < 0.01, wheat group from HFD, # *p* < 0.05, ## *p* < 0.01, ### *p* < 0.001 barley group from HFD

HFD-induced obesity is associated with increased liver triglycerides and lipogenic enzyme expression. Both flour supplemented diets significantly decreased liver triglycerides (Fig. 6A) and reduced expression of key lipogenic enzymes in the liver and adipose tissue compared to HFD control rats. Specifically, WB supplementation decreased hepatic gene expression of *Acc* and decreased adipose gene expression of *Fasn*, *Pparg*, and *Acc*, whereas BF supplementation decreased hepatic gene expression of *Fasn* and *Pparg* and decreased gene expression of *Fasn*, *Scd1*, and *Acc* in the adipose tissue compared to HFD controls (Fig. 6B). Additionally, liver triglyceride content was positively correlated with hepatic *Fasn* (R^2^ = 0.239, *p* < 0.01) and *Acc* (R^2^ = 0.306, *p* < 0.01) gene expression but not *Pparg* (R^2^ = 0.116, *p* < 0.082) (Additional file 1: Figure S4A-C), and adipose tissue mass was positively correlated with adipose *Acc* (R^2^ = 0.191, *p* < 0.05) gene expression but not *Pparg* (R^2^ = 0.021, p = 0.464) or *Fasn* (R^2^ = 0.1198, p = 0.071) across all groups (Additional file 1: Figure S4D-F). Furthermore, similar to our gene expression data in Fig. 6B, there was a non-significant reduction in protein expression of ACC in the liver of WB fed-rats (p = 0.1876) (Fig. 6C, Additional file 1: Figure S5A), while hepatic protein expression of SCD1, FAS and LPL did not differ between groups (Fig. 6D-F), Additional file 1: Figure S5B-D).

#### Wheat bran and barley flour decrease inflammatory cytokines in the liver and adipose tissue

Although WB and BF diets did not alter gut permeability in rats compared to HFD, as measured by FITC-dextran assay (Additional file 1: Figure S2A), an increase in portal endotoxin levels was observed in WB-fed rats compared to HFD-fed rats (Additional file 1: Figure S2B). Despite the increase in portal endotoxins, WB-fed rats had reductions in TNF- α expression in the liver compared with HFD controls, and both WB and BF diet groups demonstrated a non-significant decrease in IL-6 expression in adipose tissue compared with HFD-fed rats (Additional file 1: Figure S2C).

#### Plant flour supplementation alters the cecal gut microbiota and portal bile acids

**Fig. 7:**
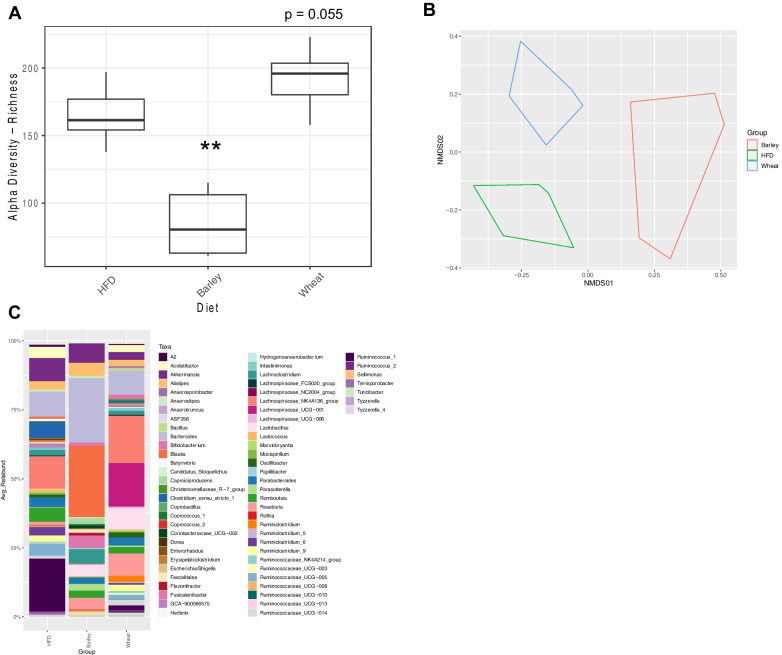
Cecal microbiota analysis. Alpha diversity index ASV/Species Richness. Statistically significant differences between all diet groups were determined using a Kruskal–Wallis test followed by a pairwise Wilcox test with Bonferroni correction (**A**); *represents significance between diet groups and HFD (**p < 0.01). Non-metric multidimensional scaling (NMDS) using Bray–Curtis dissimilarity where each dot represents one animal (**B**). Stacked barplot showing the composition of the microbial community at genus level for each of the analyzed groups (**C**). N = 8–10 per group in all panels

Rats fed a diet supplemented with BF had significantly decreased cecal microbiota alpha diversity compared with HFD-fed control rats whereas rats fed a diet supplemented with WB had a non-significant increased cecal microbiota alpha diversity (p = 0.055) (Fig. 7A). Cecal microbiota beta diversity was significantly different between rats in all diet groups (Fig. 7B, C). At the genus level, DESeq2 analysis demonstrated that WB supplemented rats had increased *Lachnospiraceae_UCG001* and decreased *Ruminococcus_1* abundance compared with the HFD-fed rats, while the BF supplemented rats had increased *Blautia* and *Lachnoclostridium* abundance compared with HFD rats. Both flour supplemented diet groups had increased abundance of *Lactobacillus* compared with HFD rats (Additional file 1: Figure S3A, B), similar to Experiment 1. The changes in the cecal microbiota composition were accompanied with a significant increase in cecum mass in the BF rats (9.9 g + 0.86 versus 4.1 g + 0.33 for HFD and 4.33 g + 0.27 for WB).

**Fig. 8:**
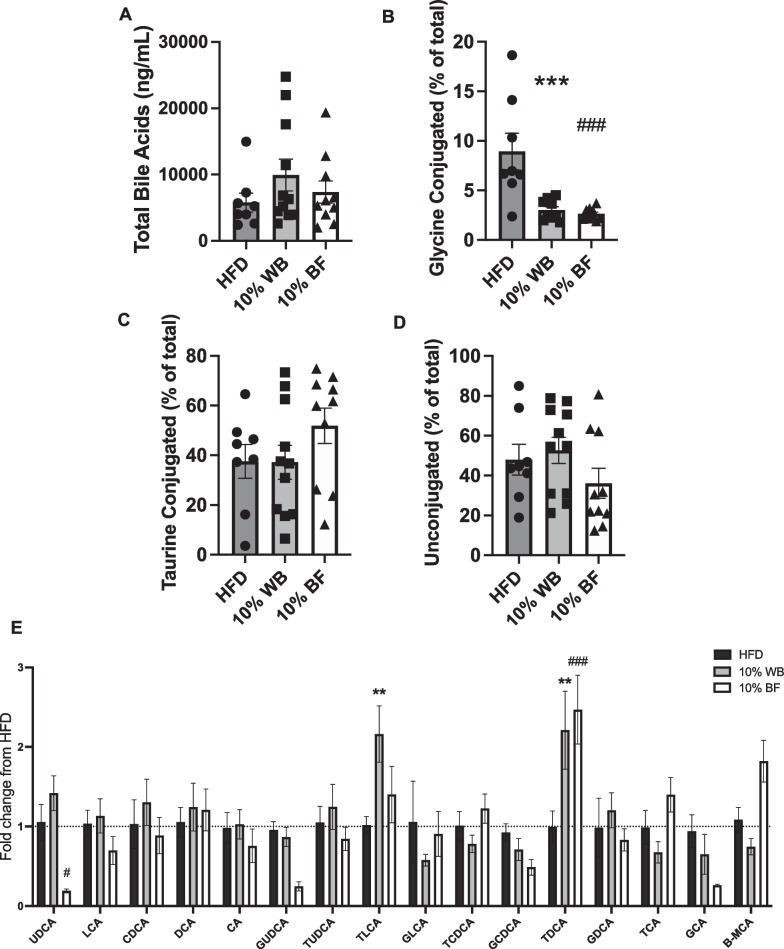
Effect of flour supplementation on portal vein bile acids. Portal vein total bile acids (**A**), % glycine conjugated bile acids (n = 8–10 per group; outliers 3 or more standard errors from the mean were removed) (**B**), % taurine conjugated bile acids (**C**), and % unconjugated bile acids (**D**). Fold change of primary and secondary bile acids over HFD control group (**E**). Data presented as mean ± SEM (n = 8–11 per group); **p < 0.01, ***p < 0.001 wheat group from HFD, #*p* < 0.05, ###*p* < 0.001 barley group from HFD

While no differences in total bile acids were found between groups (Fig. 8A), both WB and BF supplementation significantly decreased the percentage of glycine conjugated bile acids compared with the HFD group (Fig. 8B). No differences were found between groups for percentage of taurine conjugated (Fig. 8C) or unconjugated bile acids (Fig. 8D). WB significantly increased the amount of taurolithocholic acid (TLCA), BF significantly decreased the amount of ursodeoxycholic acid (UDCA), and both diets significantly increased taurochenodeoxycholate (TCDA) (Fig. 8E).

## Discussion

Increasing general fiber consumption can prevent or slow the development of obesity and obesity-related metabolic perturbations [40, 41]; however, no study to date has comprehensively compared the effectiveness of different plant-based flours that vary in fiber composition. In the current study, we found that 10% WB and BF diets were effective at attenuating adiposity gain in a diet-induced obese rodent model as well as preventing against the development of obesity and metabolic impairments commonly seen with HFD feeding. BF is high in soluble β-glucan, and the beneficial effects of β-glucan supplementation have been extensively reported [26, 42–44]. WB, on the other hand, is mostly composed of insoluble fiber. Given that both flours were effective despite being different solubilities, fiber solubility may not be imperative for fiber-mediated improvements in metabolic parameters, but improvements may be due to alterations in the gut microbiota and metabolites instead.

In the current study, HMA (5% or 10%) and OB (5%) displayed worsened body weight and adiposity compared to the HFD-fed rats. In support of these findings, previous studies have shown HMA does not improve body composition [45–47]. This could be due to the lack of increases in butyrate production with HMA supplementation, as the current study demonstrates that the effective flours, WB and BF, drastically increased butyrate production. The 5% OB diet also failed to improve metabolic parameters, despite increasing butyrate concentration similarly to rats supplemented with 5% WB and 5% BF diets. As supplementation with the 5% OB, WB, and BF diet formulations failed to improve body weight and adiposity measurements but supplementation with the 10% WB and BF diet formulations improved body weight and adiposity measurements and had the highest concentrations of butyrate, it is plausible that there is a minimum threshold for the beneficial effects of butyrate that was not reached by supplementation with the 5% flour formulations. Likewise, it is possible 10% OB supplementation may have been effective considering OB is similarly high in β-glucan to BF, a hypothesis supported by previous studies [48]. However, formulation of a 10% OB diet was not feasible in this study, therefore, the effectiveness of a higher amount of OB to improve obesity was not investigated. It should be noted that supplementation with highly viscous fibers and flours, such as β-glucan and BF, can increase small intestinal viscosity, resulting in improvements in glucose homeostasis and reductions in hepatic lipid accumulation in HFD-fed rats [49, 50]. However, these studies report no reduction in hepatic FAS gene expression, which was found in our study [49, 50], indicating that the beneficial effects of BF in this study cannot be attributed solely to increases in small intestinal viscosity. Rather, the gene expression reductions in hepatic FAS may be secondary to reductions in body weight and adiposity [51, 52] or due to other observed changes, such as SCFAs that are absorbed into circulation and readily taken up by the hepatocytes [53, 54]. Importantly, butyrate, which was significantly increased upon BF supplementation, is a known histone deacetylase inhibitor and has been reported to alter both gene and protein expression in the liver to improve lipid metabolism [55, 56]. These data taken together suggest that the type of fiber as well as the source of the fiber can influence its effectiveness.

We found that at 3 weeks of dietary intervention, WB and BF supplemented rats had a reduction in total caloric intake and BF supplemented rats had increased energy expenditure during the dark cycle. As this reduction in energy intake and increase in energy expenditure preceded reductions in body weight and adiposity, it is plausible they contributed to the observed improvements in adiposity and body weight. This reported decrease in energy intake is in line with previous studies [43, 57, 58]. Interestingly, we also found that the rats supplemented with WB had reductions in meal size but increased meal number compared with HFD-fed control rats, indicating that the rats supplemented with WB had improvements in satiation but not satiety. These findings are in line with previous research reporting increased satiation with WB preload [59] and that supplementation with a fiber derived from WB decreases body weight gain over time [22] but does not affect satiety [60]. While decreased caloric intake could be attributed to increased gut peptide signaling which is known to reduce food intake, we only observed an increase in portal levels of PYY in WF-fed rats. However, given that both GLP-1 and PYY are released in response to a meal, future studies should assess postprandial levels of these gut peptides following WB and BF supplementation. Interestingly, previous studies have demonstrated that reductions in body weight following fiber supplementation are associated with increased energy expenditure [42, 61, 62]. However, in the current study neither WB nor BF supplementation affected energy expenditure.

Despite the differences in the effectiveness of dietary-flour supplementation on energy homeostasis, all flour-supplemented diets significantly shifted the gut microbiota composition compared with HFD-fed controls. However, the shifts in the gut microbiota composition were unique for each flour. Groups supplemented with OB, BF, and WB, but not HMA, had increased relative abundance of *LachnospiraceaeUCG001* and *Lactobacillus* compared with HFD-fed controls. Both genera have been previously reported to improve gut barrier integrity and produce SCFAs, thereby reducing inflammation [63–66]. Interestingly, the same increase in *LachnospiraceaeUCG001*, in WB supplemented groups, and *Lactobacillus* in both WB and BF supplemented groups occurred in experiment 2, indicating that flour supplementation results in similar gut microbiota alterations regardless of when supplementation is initiated. Importantly, 5% OB supplemented groups had increased *Lactobacillus* and *LachnospiraceaeUCG001* relative abundance similar to 5% WB and 5% BF supplemented groups but did not improve metabolic parameters, a further indicator that supplementation with a 10% OB diet, if it could have been generated, could improve adiposity and glucose homeostasis similar to what was observed with 10% WB and 10% BF supplemented diets. OB and BF are both high in the fiber β-glucan, which could explain the similarities between 5% OB and 5% BF supplementation. Additionally, *Ruminococcus_1* was decreased in all flour supplemented groups except for the OB supplemented group compared with HFD-fed controls, and we found that *Ruminococcus_1* was negatively associated with cecal levels of butyrate and propionate. *Ruminococcus_1* is associated with mucosal layer defects in genetically obese mice [67], implicating their importance in regulating the gut barrier, and thus, inflammation. Additionally, *Blautia*, which has been shown to be decreased in individuals with obesity and insulin resistance [68, 69], was increased in the 10% BF supplemented groups in both experiment 1 and experiment 2. *Blautia* is a SCFA-producing bacteria [70], indicating that increases in *Blautia* abundance could be contributing to the increased butyrate seen with BF supplementation and the resultant beneficial effects of BF supplementation on energy and glucose homeostasis. Indeed, *Blautia* was positively associated with cecal butyrate levels. However, since 10% BF supplementation decreased body weight in both experiment 1 and experiment 2, the increases in *Blautia* abundance could be due to reductions in body weight or food intake rather than directly by BF. In contrast to previous studies [71–73], we saw no increases in *Akkermansia* abundance in our flour supplementation groups. *Akkermansia* abundance is negatively associated with obesity and type 2 diabetes [74–76], and based on these data, reductions in *Akkermansia* in the HMA supplemented groups may be at least partly contributing to the obesogenic effects of this diet. Furthermore, *Roseburia* is a known butyrate-producer that is associated with metabolic benefits [77, 78]. Interestingly, we observed an increase in relative abundance only in the 5% OB group, further highlighting that if we were able to formulate a 10% OB supplementation, we might have observed metabolic improvements with 10% OB supplementation due to increased butyrate production. Interestingly, *Lachnoclostridium* relative abundance was also increased in the BF group, despite previous studies showing *Lachnoclostridium* is positively associated with obesity and adiposity [79] and is decreased during calorie restriction [80]. Since BF supplementation led to robust benefits to energy and glucose homeostasis, the increase in *Lachnoclostridium* abundance observed with BF supplementation could be offset by other beneficial alterations to the gut microbiota or this genus may play a less significant role in metabolic homeostasis, however this remains to be tested. Furthermore, the alterations to the gut microbiota are conserved between experiment 1 and experiment 2, highlighting that the flour composition, rather than supplementation timing, plays a larger role in shifting the gut microbiota. However, since this study used terminal timepoints for the gut microbiota analyses, future studies should investigate whether the gut microbiota changes occur prior to changes in body weight or adiposity, or are a consequence of the improvements in bodyweight and adiposity.

Some of the flour-induced changes in the gut microbiota composition were associated with increased cecal butyrate, such as *Blautia* and *Ruminococcus_1*. The increases in cecal butyrate found with almost all flour supplementations aligns with previous studies which consistently report the largest increase in butyrate rather than acetate or propionate [81–85] after fiber supplementation. Interestingly, HMA supplementation, which worsened body weight and adiposity gain, increased cecal propionate levels but did not increase cecal butyrate levels, indicating that the composition of the SCFA pool may be more important for metabolic improvements than increases in total SCFA production [86]. 10% WB and 10% BF supplementation conferred the greatest metabolic improvements and caused the largest increase in cecal butyrate concentrations, indicating that increased butyrate could be mediating the improvements in metabolic homeostasis observed with flour supplementation. Of note, although not significant, there appeared to be a dose–response of fiber supplementation and butyrate concentrations in the rats supplemented with WB or BF. Additionally, this dose response might be underrepresented due to the fact that we sampled the cecum after a 5 h fast, while it has been previously shown there is a very significant postprandial rise in cecal butyrate levels up to eight hours [30]. Further, rats supplemented with 5% OB had increased cecal butyrate levels similar to rats supplemented with 5% WB and 5% BF. Therefore, again, it is likely that 10% OB supplementation, as it has previously been shown to confer metabolic improvements and increase cecal butyrate levels [48], would have led to beneficial effects similar to what was observed in the 10% WB and 10% BF supplementation groups.

SCFAs act as ligands on the G-protein coupled receptors, FFAR2 and FFAR3, localized on EECs in the distal gut to induce release of GLP-1 and PYY that regulate glucose homeostasis and overall energy homeostasis [87]. While we only observed minimal differences in gut peptides, both GLP-1 and PYY are nutrient-induced satiation signals increased in response to a meal, and it is possible postprandial increases in SCFAs [30] could lead to greater circulating GLP-1 and PYY levels in the flour-treated groups compared with the HFD group. Additionally, butyrate is the main energy source for colonocytes [88] and increases in colonic butyrate can improve gut barrier integrity [89–91] to decrease circulating endotoxin levels [91, 92]. Interestingly, despite observing little effect on gut permeability or circulating endotoxin levels, BF and WB supplemented rats had decreased cytokine expression in the liver and adipose tissue compared to HFD. While this lack of improvement on gut barrier and endotoxemia contrasts previous studies, decreased inflammatory cytokine expression in the flour-supplemented groups may be due to an increase in anti-inflammatory molecules, such as SCFAs. For example, a previous study demonstrated that treating HFD-fed mice with butyrate reduces liver and adipose inflammation without altering gut permeability [93]. Further, other studies have demonstrated that treating cells with butyrate blunts the increase in TNF- α and IL-6 expression after endotoxin stimulation [94].

Increased SCFA concentrations are also associated with decreased liver triglycerides and reductions in lipogenesis [55, 95, 96]. Indeed, in this study, we report a reduction in liver triglycerides in the flour-supplemented rats which was associated with reductions in the gene expression of the lipogenic enzymes FASN and ACC but not PPAR- γ (Additional file 1: Figure S4A-C). Additionally, we found that adipose tissue mass was significantly associated with adipose tissue expression of the lipogenic enzyme ACC and there was a non-significant correlation with adipose FASN expression but was not correlated with PPAR- γ expression (Additional file 1: Figure S4D-F). FAS catalyzes the conversion of acetyl-CoA to fatty acids in de novo lipogenesis, PPAR- γ plays a key role in lipogenesis and adipogenesis, and ACC catalyzes the conversion of acetyl CoA to malonyl CoA. Taken together, these data suggest that flour supplementation led to reductions in liver triglyceride formation by reducing lipogenesis in the liver and adipose tissue. Indeed, although not significant, we found that lipogenic protein expression in the liver was similar with our gene expression data, suggesting that the flour-induced differences in gene transcription resulted in functional protein differences to alter hepatic lipogenesis. This provides evidence that flour-supplemented diets could also treat non-alcoholic fatty liver disease, the most common type of chronic liver disease, often found as a comorbidity in individuals with obesity [97]. Furthermore, WB supplementation reduced RER during the dark cycle, indicating that there was a shift towards more lipid oxidation in this group. Taken together, reductions in lipogenesis and increases in lipid oxidation could mediate the reductions in body weight and adiposity during plant flour-supplementation.

In the current study, BF and WB supplemented groups demonstrated improvements in glucose tolerance which was accompanied by an improvement in insulin tolerance in the BF supplemented group. Taken together, this indicates that the improvements in glucose homeostasis seen with BF supplementation are likely due to improvements in insulin sensitivity. As such, future studies should determine how flour supplementation can improve insulin sensitivity. One mechanism may be through the observed reductions in overall adiposity and hepatic triglycerides or inflammatory signaling at metabolic tissues following BF and WB supplementation, as increased lipid accumulation and increased inflammation are known to cause insulin resistance [98, 99]. Secondly, exogenous butyrate administration improves glucose tolerance in rodents [100], therefore, high butyrate levels in rats fed BF and WB supplemented diets could be driving the improvements in glucose tolerance. Third, improvements in glucose homeostasis may be due to gut microbiota-induced alterations in bile acid signaling [101]. Although there were no differences in total bile acids in the current study, flour supplementation resulted in alterations to the bile acid pool. WB-supplemented groups had increased plasma levels of TLCA and TDCA while the BF group had increased plasma levels of TDCA. TLCA and TDCA are TGR5 agonists [102, 103], and TRG5 agonism improves energy and glucose homeostasis [104, 105], indicating that flour-induced alterations to the bile acid pool could partially mediate the beneficial energy and glucose homeostatic effects of WB and BF supplementation. Importantly, a recent study found that TDCA levels are decreased in diet-induced obesity and restored with gastric bypass surgery-induced weight loss [106]. Furthermore, treatment with TDCA significantly reduced body weight in diet-induced obese mice but not chow fed controls [106], indicating that reductions in TDCA may contribute to the metabolic perturbations of HFD and that the beneficial effects of flour supplementation seen in this study may be partially due to restoration of TDCA levels. Future studies should investigate this hypothesis and the role of bile acid signaling in the effects of flour supplementation on metabolic parameters.

## Conclusions

Overall, this study comprehensively investigates the effectiveness of supplementing various flours at different concentrations on metabolic homeostasis. Importantly, we found that 5% flour supplementation had no therapeutic effect to decrease adiposity in diet-induced obese rats. Our data indicate that 10% WB and BF-supplemented diets are most effective at improving energy homeostasis via reductions in energy intake, and that these improvements are possibly due to gut microbiota driven increases in cecal butyrate and portal levels of TDCA. These data demonstrate the preventative and therapeutic efficacy of 10% WB and BF supplementation to abate body weight and adiposity gain and improve glucose tolerance in HFD-fed rats. These findings expand on the current knowledge of flour supplementation and identify gut microbiota-mediated mechanisms by which these flours could be exerting their beneficial effects.

### Supplementary Information

**Additional file 1.** Supplementary figures.

**Additional file 2. Supplementary Table 1.** Macronutrient and ingredient breakdown of various diets used in the study. Number in paranthesis is Research Diets diet code.

## Data Availability

The gut microbiota datasets supporting the conclusions of this are article are available in the SRA Repository (Accession Number: BioProject ID PRJNA894692).

## Abbreviations

SCFA: Short chain fatty acid
HFD: High-fat diet
WB: Wheat bran
BF: Barley flour
OB: Oat bran
HMA: Hi-maize amylose
FFAR2/3: Free fatty acid receptor 2/3
GLP-1: Glucagon-like peptide 1
PYY: Peptide YY
