# Supplementary material for: Differential effects of plant-based flours on metabolic homeostasis and the gut microbiota in high-fat fed rats
Source: Nutr Metab (Lond). 2023 Oct 19;20:44. doi: 10.1186/s12986-023-00767-8 (PMC10585811; doi:10.1186/s12986-023-00767-8)
Supplement: Supplementary file 1 — Additional file 1. Supplementary figures. [file 12986_2023_767_MOESM1_ESM.pdf]

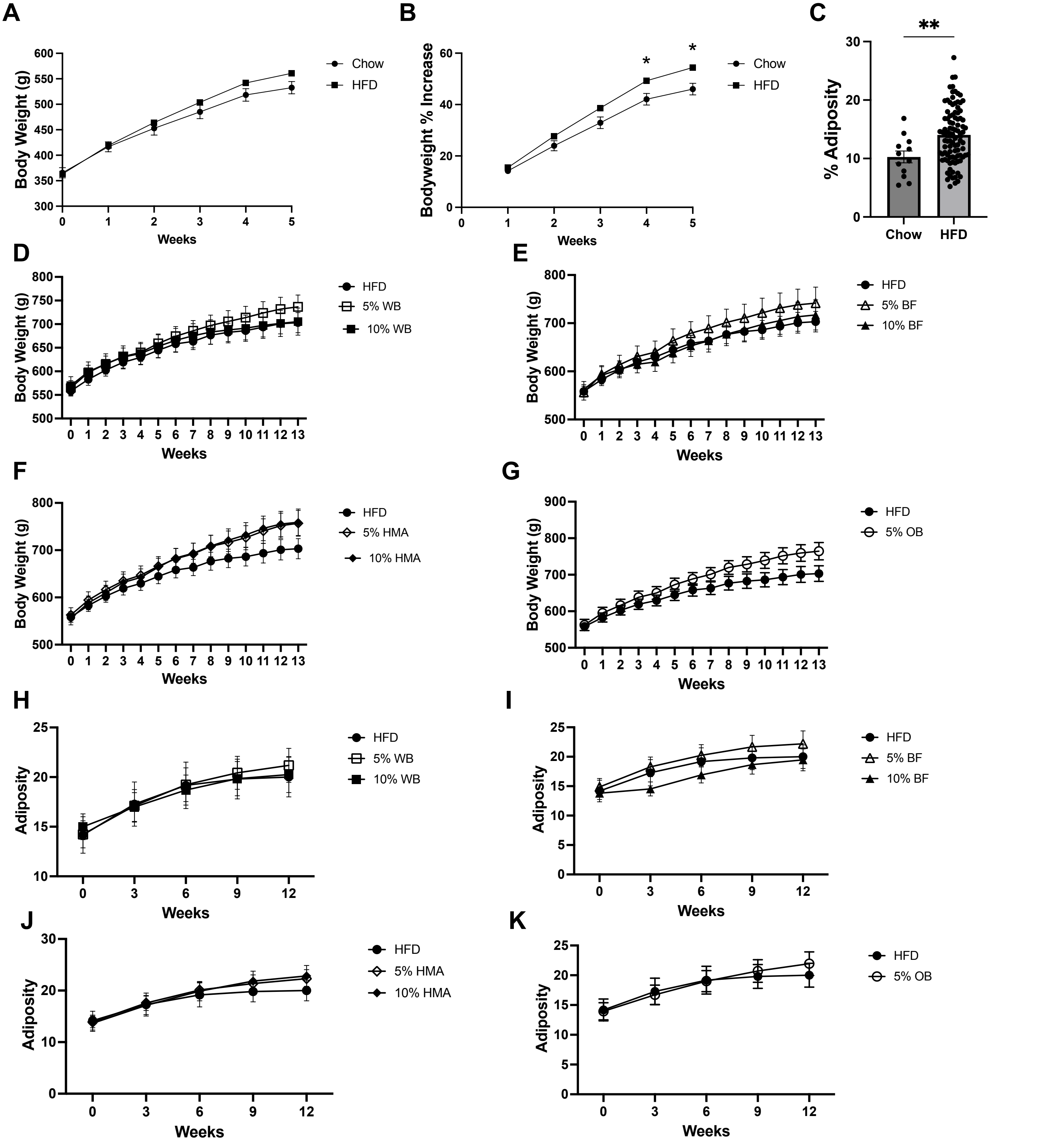

Supplemental Figure 1. 6 weeks of HFD-feeding increases body weight gain and adiposity. Body weight over time (A), body weight percent change (B), and percent adiposity at 6 weeks of HFD-feeding (C). Body weight over time for WB (D), BF (E), HMA (F), and OB (G). Adiposity over time for WB (H), BF (I), HMA (J), and OB (K). Data presented as mean ± SEM (n=12 chow, n=102 HFD); * $p < 0.05$, ** $p < 0.01$ HFD group from chow.

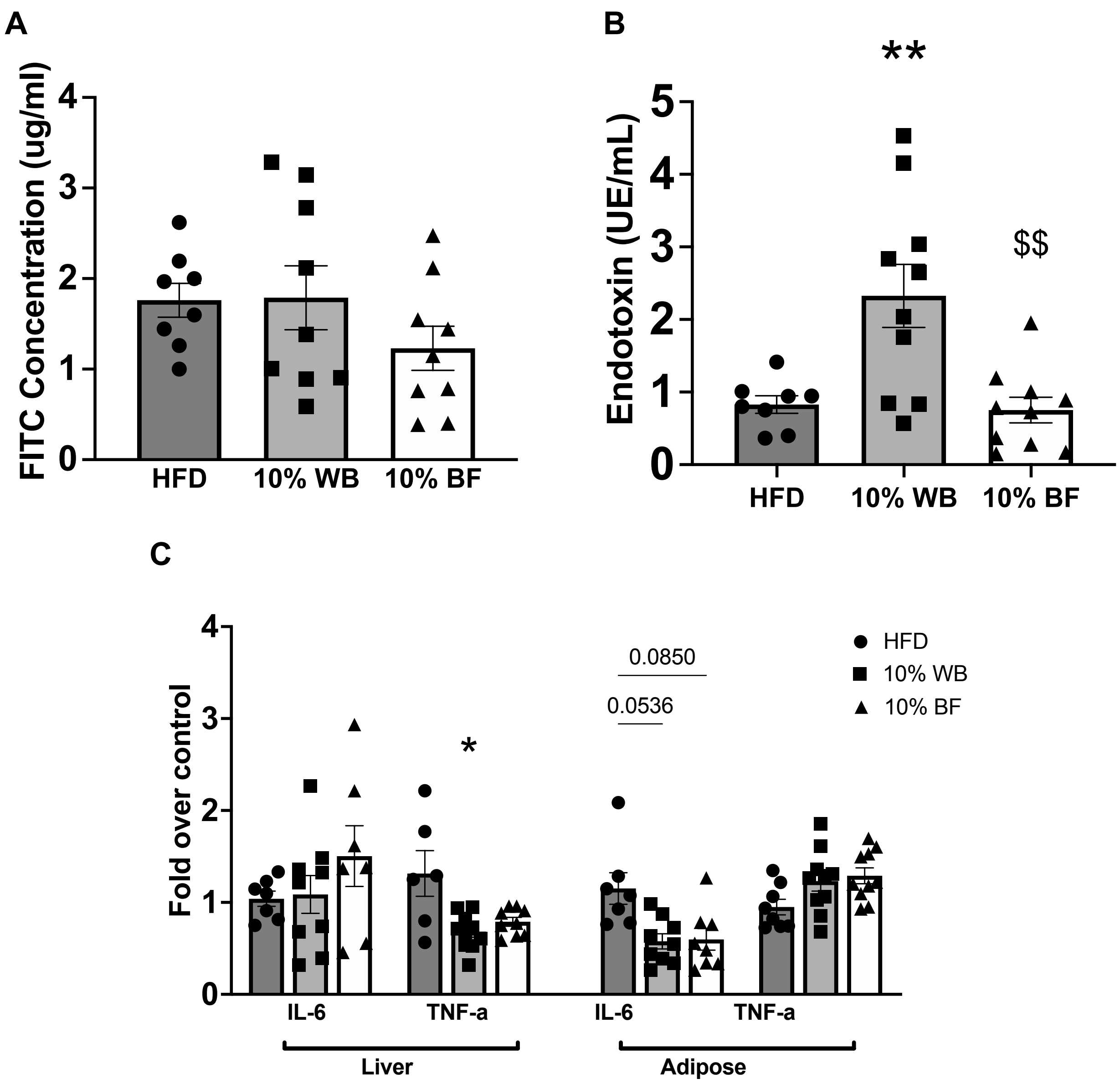

Supplemental figure 2. Portal vein FITC concentration (A), portal vein endotoxin levels (B), and liver and adipose tissue cytokine expression as fold over HFD (C). Data presented as mean ± SEM n=6-11 per group); * p < 0.05, ** p < 0.01 wheat group from HFD, $ p < 0.05, $$ p < 0.01 wheat group from barley group, trending p-values listed.

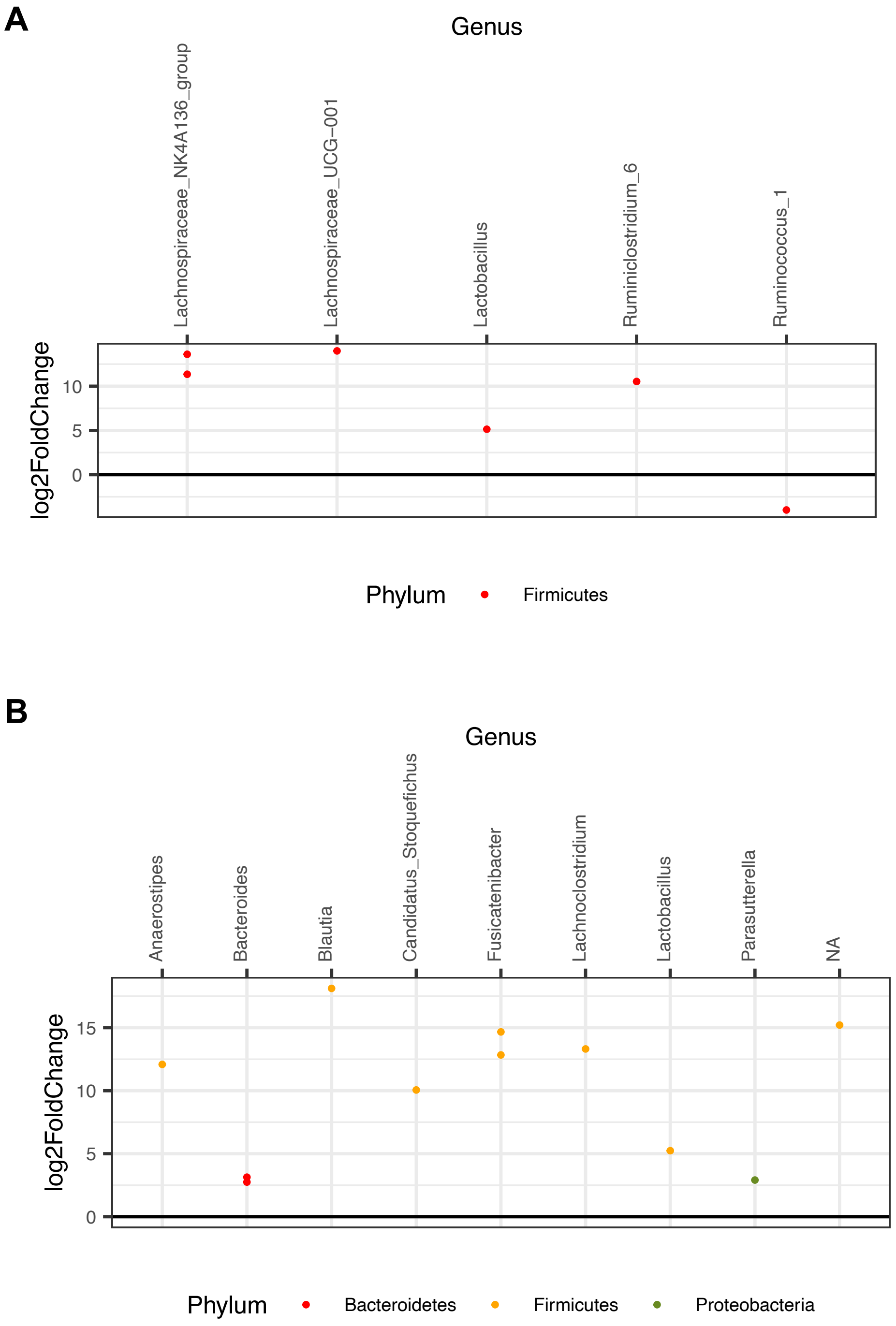

Supplemental Figure 3. Impact of flour supplementation on the cecal microbiota composition was tested using DESeq2, a differential abundance analysis tool. Each dot represents a unique ASV assigned to taxa at the genus level. If assignment to a specific genus wasn't possible, presented as NA. Colors represent specific Phylum as described below each panel. The Log2FoldChange values represent the magnitude of changes. The ASVs with positive Log2FoldChange values increased, and with negative Log2FoldChange values decreased in Barley Flour (A) or Wheat Bran (B) groups in comparison to the HFD group. The Wald test was used to identify significantly abundant ASVs between two groups.

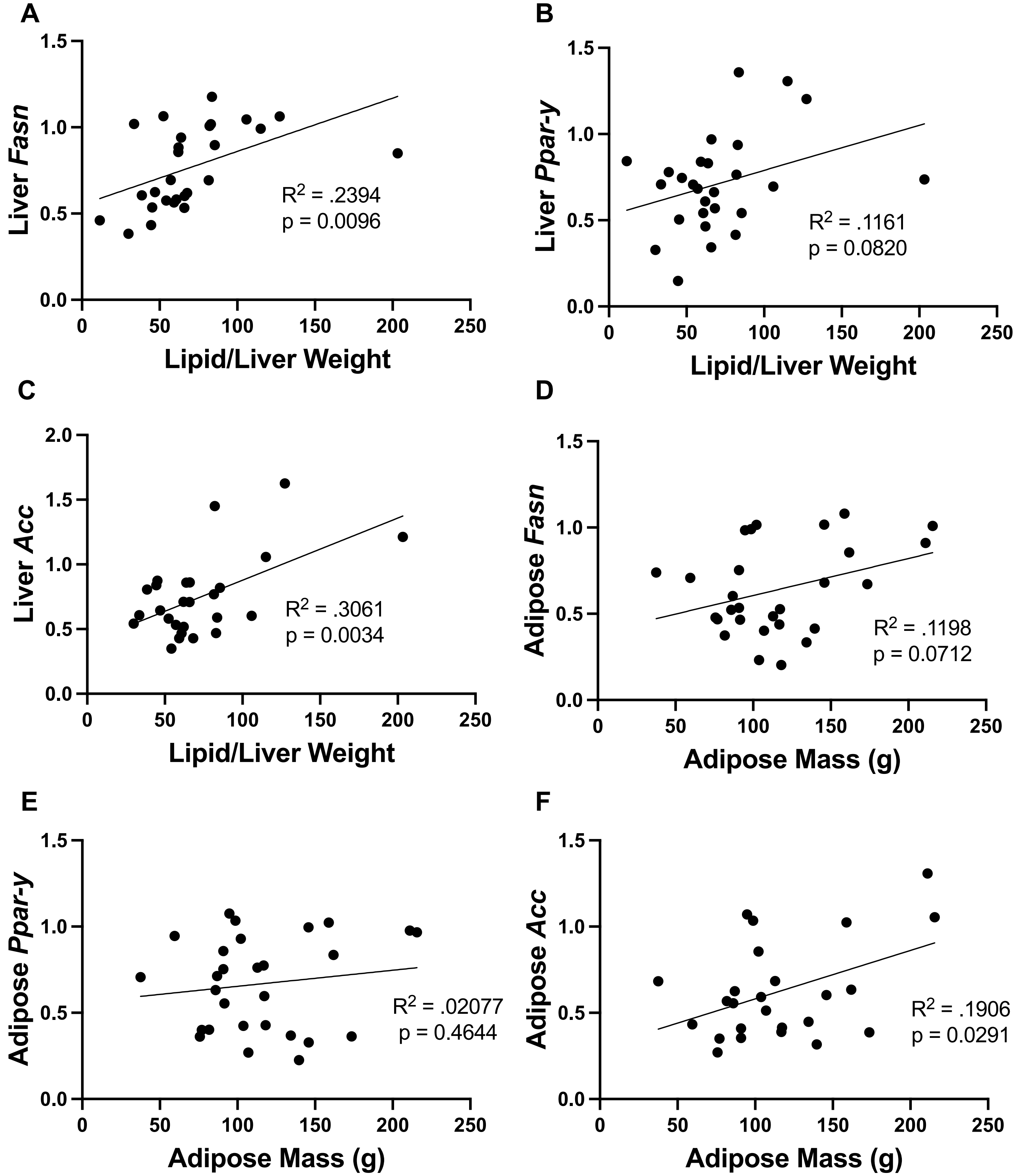

Supplemental Figure 4. Correlations between liver triglycerides and hepatic expression of Fasn (A), Ppar-y (B), and Acc (C). Associations between adipose tissue mass and adipose expression of Fasn (D), Ppar-y (E), and Acc (F).

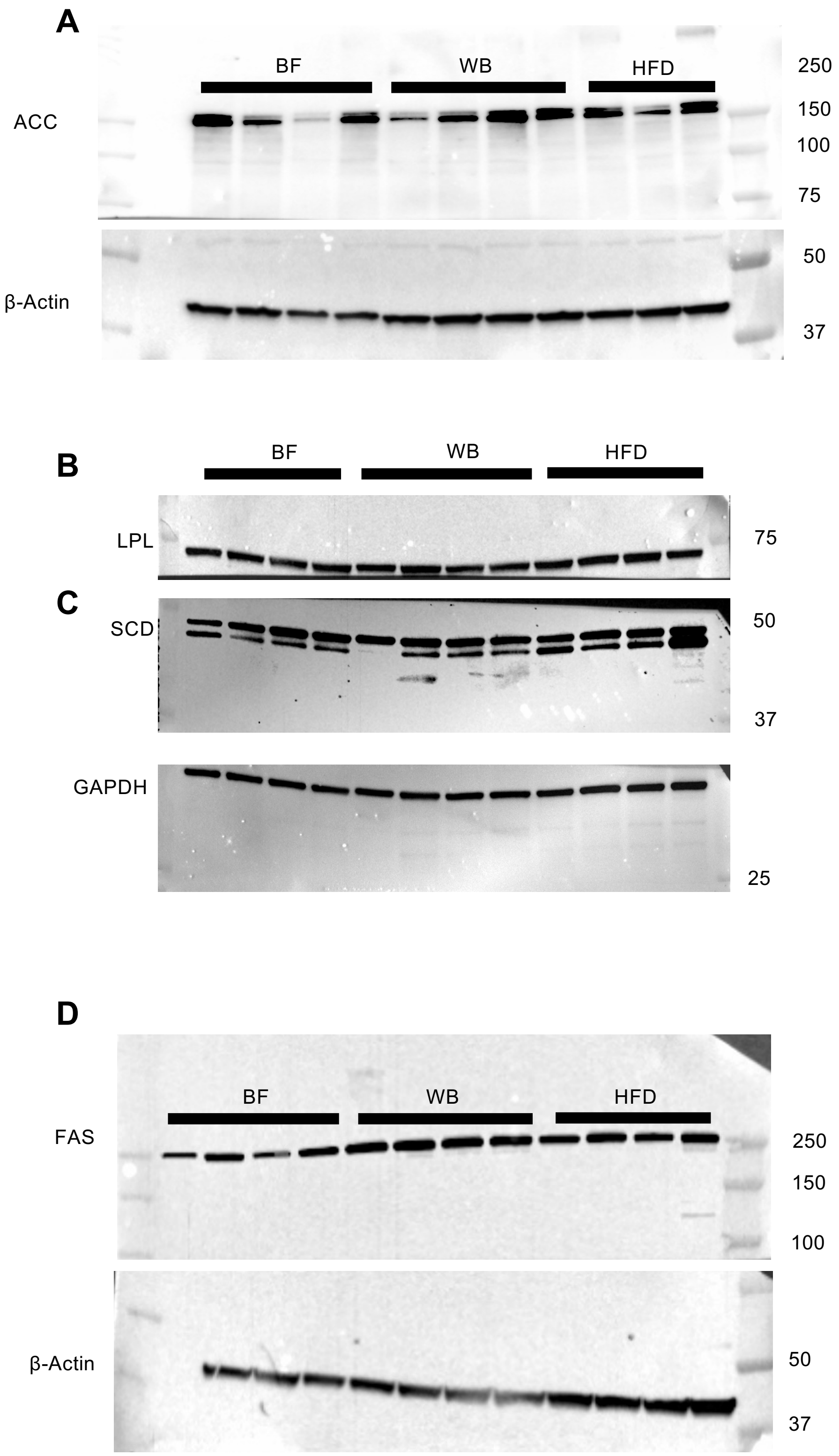

Supplemental Figure 5. Hepatic ACC (A), LPL (B), SCD (C), and FAS (D) fold control protein expression.
